# Vitamin D-Induced Molecular Mechanisms to Potentiate Cancer Therapy and to Reverse Drug-Resistance in Cancer Cells

**DOI:** 10.3390/nu12061798

**Published:** 2020-06-17

**Authors:** Mariarosaria Negri, Annalisa Gentile, Cristina de Angelis, Tatiana Montò, Roberta Patalano, Annamaria Colao, Rosario Pivonello, Claudia Pivonello

**Affiliations:** 1 Dipartimento di Medicina Clinica e Chirurgia, Sezione di Endocrinologia, Università Federico II di Napoli, 80131 Naples, Italy; negrimariarosaria@yahoo.it (M.N.); gentileannalisa58@gmail.com (A.G.); cristina.deangelis83@gmail.com (C.d.A.); tania04@live.it (T.M.); robipatalano@gmail.com (R.P.); colao@unina.it (A.C.); rosario.pivonello@unina.it (R.P.); 2 Dipartimento di Sanità Pubblica, Università Federico II di Napoli, 80131 Naples, Italy; 3 Unesco Chair for Health Education and Sustainable Development, Federico II University, 80131 Naples, Italy

**Keywords:** vitamin D, calcitriol, 1,25(OH)_2_vitamin D, colorectal cancer, prostate cancer, breast cancer, drug-resistance

## Abstract

Increasing interest in studying the role of vitamin D in cancer has been provided by the scientific literature during the last years, although mixed results have been reported. Vitamin D deficiency has been largely associated with various types of solid and non-solid human cancers, and the almost ubiquitous expression of vitamin D receptor (VDR) has always led to suppose a crucial role of vitamin D in cancer. However, the association between vitamin D levels and the risk of solid cancers, such as colorectal, prostate and breast cancer, shows several conflicting results that raise questions about the use of vitamin D supplements in cancer patients. Moreover, studies on vitamin D supplementation do not always show improvements in tumor progression and mortality risk, particularly for prostate and breast cancer. Conversely, several molecular studies are in agreement about the role of vitamin D in inhibiting tumor cell proliferation, growth and invasiveness, cell cycle arrest and inflammatory signaling, through which vitamin D may also regulate cancer microenvironment through the activation of different molecular pathways. More recently, a role in the regulation of cancer stem cells proliferation and short non-coding microRNA (miRNAs) expression has emerged, conferring to vitamin D a more crucial role in cancer development and progression. Interestingly, it has been shown that vitamin D is able not only to potentiate the effects of traditional cancer therapy but can even contribute to overcome the molecular mechanisms of drug resistance—often triggering tumor-spreading. At this regard, vitamin D can act at various levels through the regulation of growth of cancer stem cells and the epithelial–mesenchymal transition (EMT), as well as through the modulation of miRNA gene expression. The current review reconsiders epidemiological and molecular literature concerning the role of vitamin D in cancer risk and tumor development and progression, as well as the action of vitamin D supplementation in potentiating the effects of drug therapy and overcoming the mechanisms of resistance often triggered during cancer therapies, by critically addressing strengths and weaknesses of available data from 2010 to 2020.

## 1. Introduction

The term vitamin D is referred to liposoluble steroid hormones wrongly qualified as vitamins, primarily involved in calcium and phosphorus homeostasis maintenance and in the mineralization of bone [1]. Vitamin D is available in two forms: ergocalciferol (vitamin D2) and cholecalciferol (vitamin D3). Particularly, while vitamin D2 is produced in plants and yeasts and can be introduced in the human body by diet or supplementation, vitamin D3 can be either endogenously synthesized in the human skin from 7-dehydrocholesterol, upon exposure to the sun ultraviolet (UV) light, or, in a small portion, can be absorbed from the diet or introduced by supplementation [2]. However, vitamin D2 and vitamin D3 are inactive pro-hormones, which need to be hydroxylated to become active. Transported in the blood through a specific binding protein, the vitamin-D-binding protein (DBP), vitamin D2 and vitamin D3 undergo two successive hydroxylations: the first hydroxylation mainly occurs in the liver, but also in different additional tissues where enzymes with 25-hydroxylase activity are present, where the 25-hydroxyvitamin D (25(OH)D; calcidiol) is locally produced; the second hydroxylation mainly, but not exclusively, occurs in the kidney where the 25(OH)D is converted into 1,25-dihydroxyvitamin D (1,25(OH)_2_D; calcitriol) [2]. Calcitriol is the hormone active form binding to the specific nuclear vitamin D receptor (VDR), which classically regulates gene expression by binding to DNA promoter sequences [2]. In addition to these classic genomic effects, vitamin D could exert its action also by non-genomic mechanisms characterized by rapid activation of intracellular signaling molecules, which may include kinases, lipases, second messengers and Ca^2+^ and Cl^-^ channels, depending on cell type and condition [3]. In recent years, vitamin D has received much more attention given its several extra-skeletal biologic responses including inhibition of cancer cell progression, as demonstrated in different types of tumor [4]. Low levels of vitamin D have been associated with solid and non-solid cancer risk, development and growth [5,6,7,8], and vitamin D supplementation in cancer patients has been associated with a favorable prognosis [9,10,11,12,13,14]. Vitamin D anticancer actions are carried out through several mechanisms, such as inhibition of proliferation, induction of apoptosis and autophagic cell death and suppression of angiogenesis, demonstrating that vitamin D can have a crucial role in tumor suppression [15]. This strong antitumor action is probably due to the vitamin D influence on intracellular calcium oscillations, capable of influencing cell mechanisms of growth and apoptosis [16], although many other non-classical vitamin D actions unrelated to systemic calcium absorption also have a role in tumor growth inhibition [17]. Moreover, in last years, vitamin D has been shown to modulate inflammatory state of tumor microenvironment by affecting immunological infiltrations [18] and cancer stem cell proliferation [19].

More recently, accumulating evidence from epidemiological and experimental studies [9,20,21,22] has demonstrated that vitamin D, when used in combined therapy, increases the effectiveness of anticancer drugs such as gemcitabine [9], cisplatin [9], doxorubicin [21] as well as proton therapy [22] in several cancer models. In addition to synergistic or additive antitumor effectiveness induced by vitamin D supplementation, an emerging role of vitamin D as a reversal agent of drug resistance induced by chemotherapic agents [23,24] as well as target therapy [25,26,27], such as tyrosine kinase inhibitors (TKIs) [28], is spreading. The establishment of multidrug resistance mechanism is increasing in clinical practice, resulting in cancer recurrence and worsening of malignancy. To date, the molecular mechanisms by which vitamin D may reverse cancer multidrug resistance are still only partially known, and the topic is subject of a few recent studies [23,25,26,27].

The current review critically and extensively discusses the molecular mechanisms underlying the protective effects of vitamin D, the synergistic activity with different cancer drugs and the drug-resistance reversion, evaluated in in vivo and in vitro studies, on different types of solid cancer. Moreover, the review briefly reports the results of the epidemiological evidence linking endogenous levels of vitamin D and supplemented vitamin D doses to cancer risk, cancer development and growth.

## 2. Search Strategy

The relevant literature of the latest 10 years was screened from PubMed (MEDLINE), by using the following search terms: “vitamin D”, “cancer”, “solid cancer”, “colorectal cancer”, “prostate cancer”, “breast cancer”, “drug-resistance”, “reversal effect”.

Given the huge amount of epidemiological and in vivo and in vitro experimental data on vitamin D and cancer, this review mainly focuses on the role of vitamin D in a subgroup of solid tumors (colorectal, prostate and breast cancer). Conversely, considering the scarcity of studies investigating the reversal effects of vitamin D in cancer drug resistance, data available in all cancer types are presented and discussed.

## 3. Vitamin D: Synthesis and Metabolism

Circulating vitamin D derives from diet for only 20%, with the remaining 80% being synthesized in the skin where a precursor of cholesterol, 7-dehydrocholesterol, is converted to vitamin D or cholecalciferol by the UVB component of sunlight [2]. Nevertheless, the above reported percentages of circulating vitamin D levels can vary worldwide, reflecting dietary habits and supplementation, UVB exposure, which may depend on solar zenith angle (season and latitude), atmosphere condition (ozone layer and clouds) or cultural habits, and skin pigmentation [29,30]. Indeed, while western countries diet is not rich of vitamin D, vitamin D intake by diet is generally higher in North Europe and in North America countries, where the population usually consumes large amounts of oily fish and/or cod liver oil, because of large-scale vitamin D2 or vitamin D3 supplementation of a variety of food items, although the same populations are less exposed to UVB radiation, due to latitude and heavier clothes wearing. Conversely, some populations, although favorably exposed to increased UVB radiation, are frankly insufficient in vitamin D levels, due to cultural habits (full-body coverage with clothes) as for Muslims [30]. The production of vitamin D in the skin is a not enzymatic reaction, and both UVB intensity and skin pigmentation level contribute to the rate of vitamin D formation [31].

Vitamin D3, either derived from diet or produced in the skin, represents the inactive form, which requires two hydroxylations to be converted to fully active vitamin D. The first hydroxylation mainly occurs in the liver by the microsomal enzyme with 25-hydroxylase activity, CYP2R1, to obtain 25(OH)D, the major circulating, but less active, form of vitamin D [2]. Although CYP2R1 is the main hydroxylase [32], CYP27A1 or 27-hydroxylase widely distributed in several tissues with highest levels in liver and muscle, but also in kidney, intestine, lung, skin and bone [33,34,35,36], and additional enzymes with hydroxylase activity may affect the local levels of 25(OH)D [2].

A second hydroxylation occurs in the kidney and peripheral tissues by the only enzyme with 1α hydroxylase activity, CYP27B1, which transforms 25(OH)D in 1,25(OH)_2_D, the fully active and most potent metabolite of vitamin D, commonly called calcitriol. The 1α hydroxylase is expressed in the kidney, skin, brain, as well as in placenta, testis, intestine, lung and myeloid cells [37,38]. In particular, in the latter, the 1α hydroxylase expression was reported to be modulated by interferon (IFN) gamma and Toll-like receptor (TLR) [39].

Circulating levels of 1,25(OH)_2_D and 25(OH)D are finely controlled by the balance between production and degradation processes, with the aim to maintain their circulating values into physiological normal levels. Vitamin D physiological status is defined when 25(OH)D circulating levels are between 25 to 70 ng/mL. The enzyme with the 24-hydroxylase activity, CYP24A1, catalyzes the degradation reactions of 25(OH)D and 1,25(OH)_2_D.

By multiple hydroxylation reactions on the side chain, CYP24A1 catabolizes 25(OH)D, to prevent its eventual activation in 1,25(OH)_2_D, and/or 1,25(OH)_2_D, for which CYP24A1 has higher affinity compared with 25(OH)D, to stop its biologic activity [2,40].

Calcium requirement is one of the factors involved in the regulation of circulating levels of vitamin D to maintain the correct skeletal integrity. High serum calcium levels suppress renal 1-hydroxylase, while low serum calcium levels stimulate parathyroid hormone (PTH) secretion from parathyroid glands [41]. PTH acts on the kidney inducing renal 1α hydroxylase activity, therefore stimulating 1,25(OH)_2_D production [2]. Conversely, with normal level of calcium, 1,25(OH)_2_D downregulates PTH secretion, inhibits renal 1α hydroxylase activity concurrently inducing the 27-hydroxylase. Increased serum calcium and phosphorus levels can antagonize vitamin D action by inducing its degradation via 24-hydroxylase [42].

Since the important role of 24-hydroxylase in 1,25(OH)_2_D levels and half-life control, it is also considered an oncogene, as demonstrated in several solid cancers, including colorectal [43], prostate [44] and breast [45] cancer.

## 4. Vitamin D Mechanisms of Action

1,25(OH)_2_D is a steroid hormone with a wide range of physiological functions performed by binding specific receptors [46,47,48]. The 1,25(OH)_2_D actions can be divided in classical (genomic actions) and non-classical (non-genomic) actions. Genomic and non-genomic actions of 1,25(OH)_2_D are mediated by classical nuclear and non-classical membranous VDR, respectively [2].

VDR, an intracellular receptor belonging to the nuclear receptor superfamily of ligand-activated transcription factors, is classically associated with calcium and phosphorus homeostasis [47]. Upon binding to its ligand, VDR translocates from the cytoplasm into the nucleus, where together with the nuclear accessory factor retinoid X receptor (RXR), expressed in three different subtypes (RXRα, RXRβ and RXRγ) [49], binds to vitamin D response elements (VDREs) on DNA, resulting in the activation of gene transcription [50]. Although a high variability exists between the different VDR responsive elements sequences, the highest affinity for VDR is shown by the so-called motif DR3, characterized by repeats of hexanucleotides with three nucleotides spacing [51]. Moreover, recent evidence highlighted that VDREs are not associated with VDR-mediated repression of gene transcription [52]; it has been demonstrated that VDR may form complexes with different transcriptional factors to repress gene transcription [52], by regulating immune and oncogenic pathways. Moreover, it is well recognized that 1,25(OH)_2_D is able to activate non-classical, non-genomic effects, which are not mediated directly by nuclear VDR–ligand and DNA interaction, but dependent on protein–protein interaction mechanisms activating intracellular molecules. Among these molecules, the adenylyl cyclase/cyclicAMP/protein kinase A (AC/cAMP/PKA) system and a phosphoinositide-specific phospholipase C (PLC) trigger the rapid generation of intracellular second messengers [3]. In fact, the activation of PLC triggers by diacylglycerol (DAG) and inositol-1,4,5-triphosphate (IP_3_), promote the activation of protein kinase C (PKC) and rapid release of Ca^2+^ from the endogenous stores, thus controlling a wide range of intracellular physiological mechanisms [53].

## 5. Vitamin D and Cancer: Epidemiological Evidence

As largely demonstrated, VDR is not only present in cells and tissues involved in calcium regulation, but it is also expressed in malignant cells, by suggesting a pivotal role of vitamin D in cancer growth and progression [54,55]. Several observational studies have shown that circulating levels of 25(OH)D, used as surrogate marker for the evaluation of vitamin D status, may be associated with cancer risk [5,6,56,57,58,59] and prognosis [6,60,61,62,63,64,65]. A strong association between low 25(OH)D circulating levels and colorectal [6,56,60,61,66,67,68,69,70,71,72], prostate [59,62,63,73,74,75,76,77,78,79] and breast [52,58,80,81,82,83,84,85,86,87,88,89,90,91,92,93,94] cancer risk has been reported.

Colorectal cancer, particularly in late stages, is associated with vitamin D deficiency [56,66,67,68,69], also related to 1α-hydroxylase downregulated expression, which is strongly correlated with cancer severity [95] whereas higher circulating levels of 25(OH)D are associated with a decreased risk of colorectal cancer in both Asian (OR: 0.75; 95% CI: 0.58 to 0.97; *p* = 0.034) [70] and Western populations (*p* = 0.041) [71].

The relationship between vitamin D and prostate cancer risk is still controversial, due to contradictory data reporting an inverse correlation [62,73,76,79,96], or a direct correlation [78,97], or even the absence of any correlation [74,75,77] between 25(OH)D levels and prostate cancer. Interestingly, a recent meta-analysis of 21 observational studies suggests a negative role of vitamin D in prostate cancer, by reporting a direct correlation between high 25(OH)D levels and prostate cancer risk (OR: 1.17, 95% CI: 1.05 to 1.30, *p* = 0.004), therefore inviting to caution in vitamin D supplementation [97]. Discrepancies in the investigated cohorts’ characteristics, such as age, BMI, baseline vitamin D status, the dosage of serum vitamin D levels in pre and post diagnosis phase or in different stages of the disease, as well as the presence or absence of adjustment for potential confounding factors, might contribute to inconsistency among studies and to a challenging interpretation of data. However, recent findings demonstrated that isoforms of VDR [98], vitamin D-metabolizing genes [99,100] and DBP [6,101] may be associated with prostate cancer risks or clinical outcomes. The inconsistent findings on the role of vitamin D in prostate cancer can be explained by the interference of insulin like growth factor (IGF) axis components, notoriously involved in prostate cancer etiology and progression [102], which may compromise the antiproliferative vitamin D action. Indeed, it has been surprisingly demonstrated that high levels of 25(OH)D, in presence of insulin like growth factor 2 (IGF2), may increase prostate cancer risk (OR:1.33; 95% CI: 1.00 to 1.65; *p* = 0.04) [103].

Similar to prostate model, conflicting data are also reported for breast cancer. As largely described, an inverse correlation between high circulating 25(OH)D levels and breast cancer risk [80,81,82,83,84,92,93] has been reported, also confirmed in postmenopausal women [85,86]. Conversely, several studies including meta-analyses of observational and case–control studies did not find any correlation between 25(OH)D levels and breast cancer risk [52,87,88,91,104]. The assessment of timing of vitamin D status, which needs to be corrected for seasonality, together with different confounding factors, may provide an alternative reading key potentially explaining conflicting data regarding the role of vitamin D in breast cancer risk. Some authors speculate that women with vitamin D deficiency during summer season were more likely to maintain deficiency during the whole year and had a higher risk of breast cancer, compared to those with vitamin D deficiency only during winter season [105]. Furthermore, the presence of VDR genetic variants was not correlated with breast cancer risk [52], although several studies evaluating the role of VDR polymorphisms in the context of cancer development revealed that FokI, BsmI, TaqI, ApaI and Cdx2, the main polymorphisms of VDR gene may contribute to influence breast cancer risk [106,107,108,109].

Besides the importance of vitamin D in cancer risk, vitamin D levels have been further investigated to explore the potential association with cancer prognosis and tumor progression, although present data do not clarify it exactly.

In patients with advanced or metastatic colorectal cancer, higher 25(OH)D circulating levels were significantly associated with higher overall survival and progression-free survival (*p* trend = 0.0009 and 0.03), respectively [6] and higher 25(OH)D circulating levels in postoperative phase were associated with a better prognosis (HR: 0.53; 95% CI: 0.33 to 0.84, *p* = 0.006/HR: 0.91; 95% CI: 0.84 to 0.99, *p* = 0.027) [60,61]. Conversely, low levels of 25(OH)D observed in colorectal cancer patients in preoperative phase were correlated to increased systemic inflammation markers such as C reactive protein (*p* = 0.0021), but not to prognosis [72]. A different condition has been framed in the prostate cancer field; higher levels of 25(OH)D in a pre-diagnostic phase were associated with an increased survival of prostate cancer patients [62], whereas in post-diagnostic phase, no association was found between 25(OH)D levels and prostate cancer prognosis [76]. Conversely, in men with localized prostate cancer with 4.4 years of follow-up, no correlation was found between circulating 25(OH)D levels, vitamin D pathway polymorphisms and prostatic serum antigen (PSA), a well-recognized marker of prostate cancer [63]. Moreover, neither advanced nor early stage prostate cancer patients pretreated or supplemented with vitamin D showed any survival improvement or evident beneficial effects [78,110].

A recent study highlighted the correlation between normal 25(OH)D levels and breast cancer patient’s survival. Patients were divided into two groups on the basis of circulating levels of vitamin D (inadequate or deficiency group vs. insufficient or sufficient group) and their survival calculated of about 60.65 months in inadequate or deficiency group compared to 76.24 months of insufficient or sufficient group [64]. Moreover, a positive association between the highest tertile (≥25.10 ng/ml) of circulating 25(OH)D levels and mortality in breast cancer has been reported by several studies [89,90,94], although a previous cohort study of breast cancer survivors did not find significant association between 25(OH)D levels and improved survival [65].

Current evidence about 25(OH)D levels and cancer considers not only the endogenous levels of vitamin D, but also the detectable circulating levels following adequate supplementation.

Due to its stability and its capability to produce higher serum 25(OH)D levels than ergocalciferol, cholecalciferol is generally used to supplement vitamin D deficiency [111].

It has emerged that cholecalciferol supplementation increased progression free survival and relapse-free survival in patients with colorectal cancer in chemotherapy regimen, even in advanced or metastatic condition, compared to patients undergoing chemotherapy or surgery alone, demonstrating that vitamin D has a crucial role in prognosis improvement [12,13,112]. In particular, the administration of high (8000 IU/d) and standard (400 UI/d) doses of cholecalciferol in combination with chemotherapy with continuous monitoring of serum calcium levels, for about 30 months, in patients with metastatic or advanced colorectal cancer, increased progression free survival compared to placebo group, reaching an objective tumor response rate of 58% in patients receiving high-dose of cholecalciferol and of 63% in patients receiving cholecalciferol standard dose [12]. Chemotherapy associated with high-dose cholecalciferol was accompanied by increased prevalence of adverse events, such as neutropenia and hypertension, compared to chemotherapy combined with standard-dose of cholecalciferol [12].

The supplementation of 2000 IU/d cholecalciferol in patients with digestive tract cancer, including colorectal cancer, did not significantly increase the 5-year relapse-free survival compared to placebo group [13]. No significant adverse events were recorded [13]. Conversely, dietary vitamin D or different dosages of cholecalciferol supplementation during the first six months after colorectal cancer diagnosis influenced 25(OH)D circulating levels, increasing its levels of about 4 nmol/L [112]. The study monitored circulating vitamin D levels at diagnosis and six months later in patients receiving chemotherapy compared to patients undergone surgery only. The results of this study showed that at diagnosis the median circulating levels of 25(OH)D was 53 nmol/L, while six months later the median circulating levels of 25(OH)D was 48 nmol/L. Moreover 20% of vitamin D sufficient (> or = 50 nmol/L) patients at diagnosis became vitamin D deficient six months later. The most consistent determinants of 25(OH)D levels over time in colorectal cancer patients were the presence of vitamin D supplementation and the type of cancer treatment. Indeed, the use of vitamin D supplements was associated with higher 25(OH)D levels and a smaller decrease in 25(OH)D levels compared to the absence of vitamin D supplements, while cancer treatment, particularly chemotherapy, was associated with a decrease of 25(OH)D circulating levels of 6.9 nmol/L compared to surgical treatment between diagnosis and six months after diagnosis [112].

Nevertheless, the only open randomized prospective study in patients with metastatic colorectal cancer showed that all enrolled patients had insufficient 25(OH)D levels. The daily 2000 IU/d cholecalciferol supplementation to chemotherapy for two years was not able to normalize circulating levels of 25(OH)D, to modify overall survival and progression free survival in colorectal patients in chemotherapy regimen [113].

As recently emerged, contradictory data are reported about the effects of vitamin D supplementation in prostate cancer progression [114]. Although calcitriol is used in the management of patients with secondary hyperparathyroidism in renal insufficiency [111], its administration at high doses has been reported in patients with prostate cancer. In detail, supplementation of cholecalciferol (ranging from 400 to 40,000 IU/die) for a period of one month in prostate cancer patients is associated with modest prostate specific antigen (PSA) and PTH reduction before prostatectomy although circulating and prostate tissue levels of 25(OH)D and 24,25(OH)_2_D significantly increased in supplemented patients from baseline as well as circulating and prostate tissue levels of 25(OH)D were significantly higher in 40,000 IU/die treated group [115]. Conversely, high doses of oral or intravenous calcitriol administration (ranging from 45 µg on day 1, 8, 15 of treatment schedule to 74 µg over 1 h, 4 to 8 h) to prostate cancer patients was not associated with clinical benefits, but to frequent episodes of high-grade toxicity such as hyperglycemia, hematological disorders and cardiac arrhythmias [116,117]. Even further, patients receiving DN-101, an oral, highly concentrated formulation of calcitriol in capsules of 45 µg combined to dexamethasone and paclitaxel showed a shorter survival rate than control group not assuming calcitriol [116]. Slight PSA variation was found in a cohort of patients receiving low doses of calcitriol (0.5 µg/kg in four doses each first day of week) [116,117]. These studies confirm the hypothesis that while lower doses of calcitriol may ameliorate clinical outcomes of prostate cancer patients, higher doses of calcitriol are not associated with beneficial effects due to their hypercalcemic side effects.

Surprisingly, in a retrospective study evaluating human epidermal growth factor receptor 2 positive (HER2+) non-metastatic breast cancer patients treated with trastuzumab from 2006 to 2012 and supplemented with low (<10,000 IU/week or 1400 IU/d) and high (>10,000 IU/peek or 1400 IU/d) doses of cholecalciferol, a significant improvement of disease-free survival has been shown, compared to the group treated with trastuzumab alone [20]. Conversely, in a group of healthy postmenopausal women supplemented with cholecalciferol 2000 IU/d and 1500 mg/d of calcium did not normalize circulating levels of 25(OH)D and no significant correlation was found between 25(OH)D levels and breast cancer development at four years. Moreover, elevated serum calcium level and renal calculi were observed as the most frequent adverse events during the study. Therefore, high calcium levels due to high 1,25(OH)_2_D stimulate 25(OH)D or 1,25(OH)_2_D degradation and, consequently, reduce rather than stimulate the beneficial effects of vitamin D [118].

Taken together, the results of epidemiological studies suggest that circulating 25(OH)D levels may be inversely associated with colorectal, prostate and breast cancer risk and prognosis, although some controversial data have been reported. Moreover, whether vitamin D supplementation may exert a beneficial role in colorectal, prostate and breast cancer progression has yet to be completely clarified.

## 6. Vitamin D and Cancer: Molecular Mechanisms in In Vivo and In Vitro Studies

Several molecular mechanisms are involved in vitamin D anticancer effects as evaluated in animal as well as in cultured cell models.

### 6.1. Animal Studies

Preclinical studies in animal models have demonstrated that vitamin D treatment may inhibit colorectal [119,120,121,122,123,124], prostate [15,125,126,127,128,129] and breast cancer [126,130,131,132] development, by reinforcing the concept that vitamin D plays a crucial role in carcinogenesis.

In a mouse model of colorectal cancer, chemically induced by azoxymethane (AOM) exposure, dietary intake of high doses of vitamin D (AIN-93G diet containing 100, 400, 1000, 2500 or 5000 IU cholecalciferol/kg diet) reduced colorectal tumor incidence, number of premalignant lesions and number of visible cancerous lesions [119]; these effects were associated with a reduced inflammatory state with a significant interleukin-6 (IL-6) messenger downregulation, when compared to mice group receiving low doses of vitamin D [121].

Interestingly, in a colorectal cancer xenograft mouse model, dietary intake of high doses of cholecalciferol increased renal expression of the vitamin D catabolizing enzyme 24-hydroxylase, by inducing significantly larger, heavier and capable to penetrate the skin tumor lesions. The addition of soy containing genistein, a natural 24-hydroxylase inhibitor, to cholecalciferol dietary scheme restores sensitivity to the antitumor effects of vitamin D [120].

Vitamin D may also act in combination with different therapies, by inducing an antiproliferative additive effect. Indeed, in both mouse and rat chemically induced colorectal cancer models (AOM-induced, 1,2-dimethylhydrazine dihydrochloride (DMH)-induced and DMH-dextran sodium sulfate (DSS)-induced) and xenografts models, vitamin D analogs, such as paricalcitol and PRI-2191, in combination with 5-fluorouracile (5-FU) or metformin, diminished the risk to develop early colon neoplasia by controlling the early stage of carcinogenesis and reducing or delaying cancer growth through a specific cell cycle arrest and apoptosis induction, when compared to the 5-FU and metformin treatment alone [122,123,124]. The reported effects were triggered by several synergistic molecular mechanisms such as antiproliferative and antiinflammatory processes. Indeed, the analysis of Wnt, β-catenin, nuclear factor kappa-light-chain-enhancer of activated B cells (NF-kB), cyclooxygenase-2 (COX-2), dickkopf-related protein (DKK-1) and cyclin dependent kinase inhibitor 1A (CDKN1A, also known as p21) messenger tissue expression revealed a significant reduction when rats with AOM-induced colorectal cancer received combined treatment of paricalcitol and 5-FU, compared to treatment with 5-FU alone [122]. Similarly, in DMH-induced and DMH-DSS-induced mouse models, the inhibition of insulin like growth factor 1 (IGF1)-mammalian target of rapamycin (mTOR) pathway was associated with the downregulation of the expression of c-MYC, a transcription factor implicated in multiple cellular processes, including proliferation, differentiation and apoptosis, and cyclin D1, an important regulator of cell cycle progression [124]. In line with these results, recent findings evaluating the association between vitamin D deficiency and tumor burden in colon cancer, demonstrated that VDR silencing attenuates vitamin D signaling in mouse cultured colonic cancer cells. The main intracellular effect was an enhanced β-catenin transcriptional activity that positively influences c-MYC messenger expression, which is often deregulated in human cancers, and notably involved in cell cycle progression, thus confirming VDR involvement in cancer growth control [133].

In prostate cancer xenograft mice receiving cholecalciferol supplemented diet, the reduction in tumor volume started to be evident after one-week treatment and became significant after three-weeks treatment, with the antiinflammatory and antiproliferative effects of vitamin D controlled by a tight gene modulation of anti-inflammatory and antiproliferative related genes, therefore upregulating COX-2, p21 and insulin like growth factor binding protein-3 (IGFBP-3) [126]. As observed in colorectal cancer models, also in prostate cancer xenograft mice, supplementation of soy to diet increased the bioavailability of endogenous or administered 1,25(OH)_2_D, and consequently its anticancer effect, although the risk of hypercalcemic adverse effects is relevant for prostate cancer [125]. The results of the study demonstrated the antiproliferative vitamin D efficacy despite the upregulation of 24-hydroxylase messenger expression levels in prostate cancer tissue. Soy diet in combination with 1,25(OH)_2_D treatment was able to achieve a further reduction of tumor growth, associated with upregulated messenger expression levels of antiproliferative (p21, IGFBP-3) and pro-apoptotic (Bcl-2 associated X; Bax) genes and downregulation of antiapoptotic (B-cell lymphoma 2; Bcl-2) and cell cycle promoting gene cyclin D1. These results confirmed that the increase of circulating vitamin D levels may control prostate cancer growth by inhibiting cell division and by inducing apoptosis [125]. In addition, the status of VDR expression may be determinant for the development of prostate cancer [15]. Using a genetically modified mouse model resuming characteristics of human prostate tissue in the early stages of tumor development, authors demonstrated that the absence of VDR was associated with larger cancer foci development [15]. Interestingly, transcriptional activity of VDR in prostate cancer tissue was strongly regulated by lysine-specific demethylase 1A (LSD1), a key regulator of the androgen and estrogen receptors [127]. As shown in prostate cancer xenograft mice, LSD1 and VDR are present in the same transcriptional complex in the nucleus, mutually interacting to regulate cancer progression through epigenetic mechanisms. LSD1, in presence of 1,25(OH)_2_D, acts as coactivator and corepressor of VDR activity thus modulating 1,25(OH)_2_D effects induced by target genes transcription [127]. Moreover, the role of vitamin D as cryosensitizer has been studied in prostate cancer xenograft models, in which pretreatment with 1,25(OH)_2_D, intraperitoneally injected 18 h before cryotherapy, caused a synergistic antiproliferative effect [128,129]. The reduction of procaspase-9 protein levels following combination treatment may suggest a substantial mitochondrial-mediated pathway in vitamin D sensitization [128,129].

The beneficial effects of dietary cholecalciferol and injected 1,25(OH)_2_D supplementation have been explored in breast cancer xenograft models [126,132]. Dietary and injected vitamin D displayed similarly efficiency with the latter having the advantage of a lack in serum calcium levels alteration, over dietary vitamin D. Dietary cholecalciferol is able to suppress estrogen synthesis and signaling in breast cancer xenograft models, and to act as antiproliferative and antiinflammatory agent [126], although these effects are often associated with an increased expression of 1α-hydroxylase messenger in the tumors and, therefore, a significant elevation in circulating vitamin D levels [132]. Furthermore, in a spontaneous non-immunodeficient model of breast cancer the exogenous administration of 25(OH)D has been shown to delay tumor progression without hypercalcemic effects [130]. In particular, while low levels of vitamin D diet (25 IU: 0.625 µg) allowed an increase in tumor growth, the continuous systemic perfusion of the 25(OH)D (2000 pmol/24 h) or 1,25(OH)_2_D (12 pmol/24) inhibited tumor growth and lung metastasis development by downregulating cell cycle progression markers expression, such as cyclin D1, without increased blood calcemia [130]. Moreover, the continuous systemic perfusion of the 25(OH)D was accompanied to increased local production of 1,25(OH)_2_D [130]. Also, 1,25(OH)_2_D seems to accelerate photodynamic therapy in a breast cancer xenograft model. Indeed, photodynamic therapy resulted more efficient in vitamin D pretreated tumors, than vehicle-pretreated tumors, therefore representing a pivotal photosensitizer for breast cancer cells [131].

Considering these in vivo studies, both dietary and injected vitamin D supplementation may reduce colorectal, prostate and breast cancer growth through several antiproliferative and antiinflammatory molecular processes, mainly involving NF-kB and COX-2 inhibition, Wnt/β catenin pathway deregulation, apoptosis induction and cell cycle arrest. Moreover, vitamin D antiproliferative effects can be potentiated by chemotherapeutic drugs and by target therapy molecules.

### 6.2. In Vitro Studies

Anticancer activity of vitamin D has been largely studied in different types of tumors, elucidating its pivotal role in cancer progression and invasion [134]. It has been reported that vitamin D inhibits colorectal, prostate and breast cancer cells growth by controlling cell proliferation [11,135,136,137,138,139,140,141,142,143,144,145,146] through cell cycle arrest [144,147,148,149,150], by regulating cell death through apoptosis [54,144,147,151,152,153] and autophagic processes [154,155], and by modulating inflammatory processes implicated in immune infiltration and tumor invasiveness [134,137,150].

Cytofluorimetric and protein analysis revealed that vitamin D-nanoemulsion causes a cell cycle arrest in G0/G1 phase in colorectal [144], breast [136,149,150] and prostate [156] cancer cells expressing VDR. The most plausible mechanism is the inhibition of cyclin-dependent kinase inhibitor, such as p21 and p27, whose expression is differently modulated by 1,25(OH)_2_D, therefore blocking the cell cycle progression in S phase in prostate [157] and breast cancer [134,151] cell lines. In both prostate and breast cancer models, 1,25(OH)_2_D exposure increased p21 messenger and protein levels [157,158]. This effect can be potentiated in case of combined therapy, as shown in prostate cancer where vitamin D and Akt inhibitor cooperate to induce a G1 cell cycle arrest and cell senescence, by inducing p21 upregulation [157]. The exact mechanism by which 1,25(OH)_2_D may regulate p21 and p27 transcription is not well clarified, although as observed for p27 transcription, the role of VDR is fundamental [159]. In prostate cancer cells, indeed, the direct interaction between VDR and specificity protein 1 (sp1) which, in turn, modulates p27 transcription, seems to be the common mechanism modulating expression of genes without VDR responsive elements [159]. The inhibition of vitamin D-mediated proliferation also passes through the control of cell death mechanisms, such as apoptosis [54,147,151,152,153] and autophagy [154,155]. In breast as well as prostate cancer cells, the long-term exposure to 1,25(OH)_2_D is able to induce apoptosis by stimulating pro-apoptotic factor Bax and Bad and by inhibiting antiapoptotic factors such as Bcl-2 [156,160], and by stimulating caspase-3 expression through the Wnt/β-catenin pathway downregulation [54]. Wnt/β-catenin pathway has also been reported to be an apoptosis mediator in colorectal cancer cells, where vitamin D-nanoemulsion treatment induced cell cycle arrest and apoptosis through β-catenin gene downregulation [144]. Moreover, the accumulation of β-catenin in the nucleus and the promotion of several oncogenes’ transcription, including c-MYC, contributes to carcinogenesis and cancer cells progression [161], since c-MYC involvement in cell cycle. Indeed, as demonstrated in several colorectal and prostate cancer cell lines, vitamin D antiproliferative effects occur by reducing c-MYC messenger and protein expression [162,163]. Therefore, 1,25(OH)_2_D inhibition of TACE (Tumor necrosis factor Alpha Converting Enzyme, also known as ADAM17), a metalloproteinase overexpressed in several cancer types [164], may further explain its antiproliferative effects [165]. ADAM17, whose expression is in turn regulated by miRNA-145 [166], enhances transforming growth factor-α (TGFα) and epidermal growth factor receptor (EGFR) expression, thus contributing to tumor growth and invasiveness [166]. It has been reported that vitamin D is able to suppress cell growth and PTH production via ADAM17 modulation [165]. Interestingly, the non-calcemic analog of vitamin D, seocalcitol (EB1089) induces a G1/S phase growth arrest, with induction of p21 expression only in breast cancer associated gene-1 (BRCA1)-expressing breast cancer cells [141]. The proposed mechanism demonstrated BRCA1 and VDR association to occupy VDR responsive elements of p21, and to promote acetylation of histone H3 and H4 [141]. Vitamin D-induced apoptotic process, characterized by caspase 3 overexpression, is a common factor in breast cancer combined therapy with tyrosine kinase inhibitors such as lapatinib and neratinib [140] or gefinitib [139], omega-3 polyunsaturated fatty acids [152] and metformin [153]. Colorectal cancer cells co-treated with 1,25(OH)_2_D and metformin showed cell growth arrest, by demonstrating the synergistic effect of the treatment and the main effect on autophagy process induction, due to an increase in microtubule-associated protein 1A/1B-light chain 3 II:I (LC3II/LC3I) ratio, the most widely used autophagosome marker, reflecting the number of autophagosomes and autophagy-related structures, in cells treated with a combination therapy compared to mono-treatments [154]. Moreover, the upregulation of the tumor suppressor beclin-1 by the chemotherapeutic vitamin D analog EB1089 has been demonstrated to be implicated in autophagy process and in lysosomal changes in breast cancer cells [155].

Considering the fundamental role of inflammation in oncogenesis [167], and that vitamin D strongly contributes to modulate cytokines and immune cells [168], several studies have been developed to investigate the role of vitamin D in inflammation processes associated with tumor development [169,170,171,172,173,174]. It is well known that a chronic inflammatory microenvironment can represent the prelude to cancer onset and, recently, scientific evidence has revealed that vitamin D can interfere at different levels in inflammatory microenvironment development and oncogenesis [168]. The involved mechanisms include the regulation of cytokines [169] and prostaglandins [170] production and the inhibition of NF-kB signaling [171,172,173,174]. In addition, while mouse embryonic fibroblasts lacking VDR showed higher NF-kB activity [171], in colon cancer cells exposed to lithocholic acid, a VDR ligand, a downregulated NF-kB activity has been reported [172].

Primary prostatic cultures of normal and adenocarcinoma cells treated with 1,25(OH)_2_D displayed lower expression levels of interleukin-6 (IL-6), accompanied to p38 MAPK inactivation [169]. Moreover, as demonstrated in human cultured macrophages treated with 1,25(OH)_2_D and stimulated with lipopolysaccharide, NF-kB activity was markedly inhibited by a significant increase of messenger and protein levels of nuclear factor of kappa light polypeptide gene enhancer in B-cells inhibitor, alpha (IkBα), the inhibitory protein of NF-kB [173]. The inhibitory effect of 1,25(OH)_2_D on IkBα phosphorylation led to IkBα half-life prolongation, which in turn can influence NF-kB nuclear translocation and, consequently, activity on stimulation of cytokines transcription such as tumor necrosis factor-α (TNF-α) secretion [173].

Similarly, human colorectal cancer cells exposed to 1,25(OH)_2_D showed antiproliferative and antimigratory effects by inhibition of TNF-α production [135], and, in MCF-7 human breast cancer cell line, 1,25(OH)_2_D influenced both IkBα expression and phosphorylation via phosphoinositide 3-kinases/protein kinase B (PI3K/Akt) pathway [174]. Moreover, the co-treatment with 1,25(OH)_2_D and TNF-α inhibited metalloprotease-9 (MMP-9) and monocyte chemoattractant protein-1 (MCP-1) protein expression, otherwise hyperactivated in presence of TNF-α alone, by demonstrating a further role of vitamin D in the regulation of infiltration, migration and cancer invasiveness [174]. Conversely, human breast cancer cells treated with 1,25(OH)_2_D showed decreased proliferation, in part induced by the reduced TNF-α and IL-1β synthesis, by demonstrating that even drastic deregulation of immune modulators may contribute to cancer progression [137].

### 6.3. Vitamin D and Cancer: A Focus on Cancer Stem Cells and miRNA In Vitro Studies

More recently, the important role of vitamin D in cancer onset and progression has been explored by studying its association with cancer stem cells [141,175,176] and a class of short (~18–22 nucleotides long) noncoding RNA, the microRNAs (miRNAs).

Cancer stem cells are a subset of cells with the characteristics of self-renewal and differentiation into multiple cell types that confer them the possibility to actively participate in tumor progression [177]. Recently, it has been demonstrated, by immunohistochemistry and RNA scope in situ hybridization, that human colon stem cells express VDR [178]. Treatment with calcitriol, indeed, in normal organoids, upregulates stemness-related genes, whereas in tumor organoids it mainly acts on cell proliferation without a significant impact on stemness-related genes expression, by demonstrating the different role of VDR in these two cell subpopulations [178]. Moreover, human colorectal cancer cells co-treated with the vitamin D analog PRI-2191 and imatinib significantly downregulated stemness-related genes expression, compared to cells treated with imatinib alone, suggesting a pivotal role of vitamin D in controlling residual colon cancer cells capable to initiate cancer recurrence [179]. Similarly, as revealed by microarray analysis in primary cultures of mouse prostate cancer, vitamin D may influence stemness-related genes expression thus contributing to cancer progression [180]. In breast cancer, treatment with a vitamin D analog, BXL1024, reduced breast cancer stem-like cells population CD44+/CD24-/low and CD49f+/CD24-/low, identified as markers of tumor-initiating cells [175]. Similarly, mammosphere or mammary epithelial stem cells formation decreased after vitamin D and BXL1024 exposure, as well as octamer-binding transcription factor 4 (OCT4) and Krüppel-like factor 4 (KLF4), stem self-renewal markers expression [176]. Moreover, two vitamin D analogs, EB1089 and QW-1624F2, reduced breast cancer cells and breast cancer stem-like cells by blocking them in G1/S cell cycle phase and by inducing p21 expression [141]. In the last years, several miRNAs involved in cancer and modulated by vitamin D have been identified [181]. miR-675–5p has been reported to regulate VDR expression in colorectal cancer cells [182], whereas miR-627 has been demonstrated to suppress mouse colorectal cancer cells proliferation via histone demethylase 1A (JMJD1A) downregulation [183], suggesting a crucial role in cancer control. Similarly, miR-125b, particularly under-expressed in malignant prostate tissue than in controls, resulted re-expressed after vitamin 1,25(OH)_2_D treatment [184]; similar findings were observed for miR-143 in prostate cancer cell line treated with 1,25(OH)_2_D [185]. Last, in MCF-7 breast cancer cells, miR-125b downregulates VDR expression, by conferring a pharmacological resistance to vitamin D [186]. Moreover, in breast cancer cell lines vitamin D stimulated the susceptibility to natural killer cells, by reducing miR-302c and miR-520c expression, demonstrating that these miRNAs are involved in susceptibility of cancer cells to immune-attack of vitamin D-dependent natural killer cells [18].

In conclusion, considering the examined in vitro studies on colorectal, prostate and breast cancer cells, vitamin D acts as anticancer agent by controlling several processes involved in cancer growth and progression. The inhibition of cancer cells proliferation, the arrest of cell cycle and the induction of cell death, such as apoptosis and autophagy, are the main molecular mechanisms by which vitamin D exerts its anticancer action. Moreover, vitamin D anti-inflammatory properties may regulate tumor microenvironment by suppressing inflammatory mediators implied in cancer invasion.

More recently, the capability of vitamin D in modulating cancer stem cells proliferation and miRNAs expression has shed new light on the vitamin D role in cancer, by underpinning regulatory properties in the stem and epigenetic fields.

## 7. Vitamin D and Cancer: Molecular Mechanisms of Cancer Drug Resistance

Different cell molecular mechanisms are involved in acquired and intrinsic resistance of cancer cells to cancer therapy. Unlike the intrinsic resistance, which appears upon the first exposure to cancer therapy, the acquired resistance occurs during the chemotherapeutic treatments or during a second attempt of treatment, after cancer recurrence [187].

In the last years, scientific evidence reported a large number of anticancer drugs capable to enhance chemotherapy or target therapy efficacy or even to overcome multidrug cancer resistance including that to xenobiotics and nanoparticles [188,189]. Recently, several studies have been carried out to investigate on the molecular mechanisms underlying chemotherapy and tyrosine kinase inhibitors cancer resistance [187]. First of all, the increased blood supply, due to the angiogenic phenomenon of metastases, could cause hypoxic areas, reported to be implied in a loss of p53-mediated apoptosis [187]. Moreover, hypoxic state is also responsible of dysregulated glycoprotein expression, which in turn, is associated with a decreased drug uptake within cells, due to a change in lipid membrane composition. Furthermore, an increased drug efflux from cancer cells has been reported, due to an overexpression of ATP-binding cassette (ABC) transporters [187]. In addition, the reduced drug influx in cancer cells does not ensure the normal interaction between drug and intracellular targets and compartments. Last, the repair of damaged DNA has a clear role in anticancer drug resistance. In particular, for chemotherapy inducing DNA damage, the activation of DNA repair mechanisms is an important survival escape carried out by cancer cells [190].

Control of cell death, abnormal functionality of cell cycle check-points and epithelial–mesenchymal transition are only a part of a wide range of proliferation and differentiation molecular events in cancer, which are implied in acquired multitarget cancer cell resistance [187]. In the specific case of target therapy, aiming at individualizing medical therapy towards precision medicine, the innate or acquired resistance mechanisms are essentially three: (1) on-target mutations; (2) activation of a parallel or downstream signaling that influence drug action; (3) activation of pro-survival pathways [191]. Equally important and of more recent interest, is the role of epigenetics which, especially in the mechanisms of resistance to target therapies, seems to be fundamental. Last, the role of noncoding microRNA (miRNAs) has been recently investigated mainly because the regions that miRNAs usually target are frequently associated with carcinogenesis [192].

In addition to all these mechanisms of drug resistance in cancer, in which vitamin D interferes at various levels, there are mechanisms of resistance to the vitamin D itself, which have also been observed in colorectal, prostate and breast cancer. In addition to the previously mentioned oncogenic activity of 24-hydroxylase, capable to control half-life of 1,25(OH)_2_D, also downregulated VDR expression may contribute to vitamin D resistance [193], as well as dysregulated intracellular effectors and transcriptional factors vitamin D-dependent [194]. However, combination of vitamin D with traditional or emerging anticancer drugs could be useful in cancer treatment.

## 8. Vitamin D and Cancer: Reversal Effects of Vitamin D on Cancer Drug Resistance

**Figure 1:**
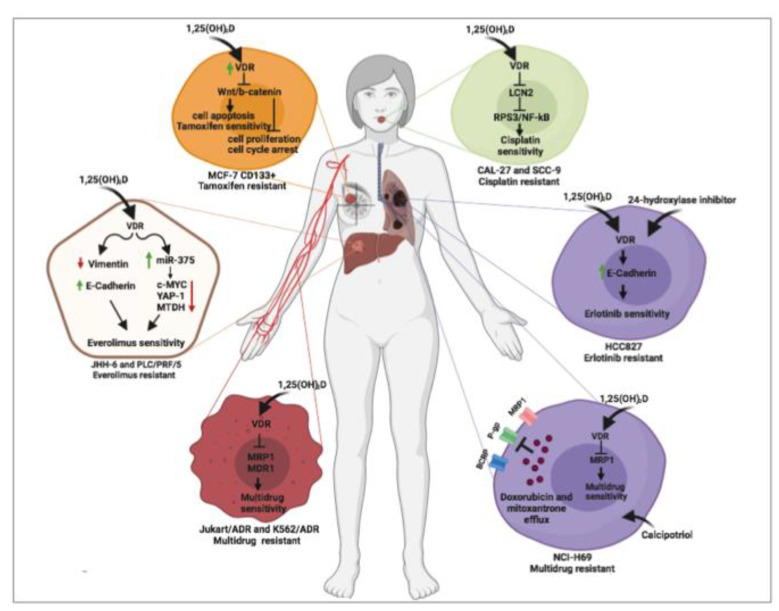
Vitamin D-induced molecular mechanisms involved in reversing drug-resistance in several tumors: oral, breast, liver and lung tumors and leukemia.

In the following paragraph, due to the scant available data, a systematic review of the current literature about the role of vitamin D to reverse drug resistance mechanisms in cancer are detailed and represented in Figure 1.

Cancer pharmacological resistance represents the major cause of tumor progression therefore, overcoming drug resistance is now the greatest challenge for scientists. Vitamin D has been reported to reverse resistance mechanisms to chemotherapy [23,24], TKI [28] and other target therapy [25,26,27], as studied in in vitro models of different cancers.

Human breast cancer cell line MCF-7 treated with 1,25(OH)_2_D, similarly to different cancer models, showed an increased percentage of apoptotic cells with a cell cycle arrest in G0/G1 phase associated with an upregulation of VDR protein expression [24]. Surprisingly, when MCF-7 stem cells were sorted using a CD133 fluorescent antibody, VDR resulted overexpressed, compared to non-stem MCF-7 cancer cells (CD133-) used as control. Moreover, VDR-overexpressing cancer stem cells developed fewer and smaller spheroids with higher apoptotic rates mediated by Wnt and β-catenin protein inhibition [24]. Last, when compared to parental MCF-7 cells, MCF-7 stem cells were more resistant to tamoxifen treatment, showing that a higher tamoxifen concentration was needed to induce MCF-7 stem cells proliferation inhibition. MCF-7 stem cells resistance was associated with a persistent activated Wnt/β-catenin pathway. Treatment with 1,25(OH)_2_D induced a VDR overexpression and inhibited MCF-7 cancer stem cells proliferation by Wnt/β-catenin proteins downregulation, demonstrating that vitamin D reverts tamoxifen resistance by inhibiting Wnt/β-catenin pathway in breast cancer cells [24]. Similarly, oral cancer cells (CAL-27 & SCC-9 cell lines) treated with vitamin D showed a partial reversion of cisplatin resistance through lipocalin-2 (LCN2) protein downregulation [23]. Chronic treatment with cisplatin enhanced the protein expression of the oncogene LCN2 by inhibiting methylation at the promoter level. Furthermore, considering that LCN2 overexpression is associated with lymph node metastasis, poor prognosis and post-chemotherapy recurrence, oral cancer cell lines overexpressing LCN2 proteins showed less sensitivity to cisplatin [23]. Surprisingly, vitamin D exposure made oral cancer cells again sensitive to cisplatin by inhibiting LCN2 expression and Nf-kB phosphorylation [23].

Moreover, reversal effect of vitamin D has been also observed in a non-small cell lung cancer cell line (HCC827^R1^ Cells) with epidermal growth factor receptor (EGFR) mutation that made these cells erlotinib-resistant. Indeed, HCC827^R1^ cells developed epithelial–mesenchymal transition (EMT) following prolonged treatment with erlotinib, an EGFR inhibitor. The switch to a more aggressive phenotype of HCC827^R1^ cells, associated with erlotinib resistance, was also characterized by the overexpression of VDR [28]. Treatment with 1,25(OH)_2_D reverted EMT and restored erlotinib sensitivity, probably by counteracting tumor growth factor β (TGF-β), which is typically induced during EMT in HCC827^R1^ cells. In this study, authors treated erlotinib-resistant HCC827^R1^ cells with liposomal nanoparticles (LP) nanocarriers systems which co-deliver vitamin D, and a 24-hydroxylase inhibitor, CTA091. This strategy, eventually translated in vivo, could avoid systemic and amplify local effects [28]. Compared to free vitamin D and CTA091, treatment with liposomal nanoparticles showed increased drug uptake within cells, significant upregulation of the vitamin D target gene pro-epithelial CDH1 and increased inhibition of colony formation in HCC827R1 cells [28].

Similarly, the reversal effect of vitamin D has been also observed in several hepatocellular carcinoma (HCC) cell lines resistant to everolimus, an mTOR inhibitor. The authors showed that the treatment with vitamin D restored sensitivity to everolimus by regulation of EMT process and miR-375/oncogenes circuit [25]. Specifically, in everolimus-resistant HCC cell lines, the treatment with vitamin D induced EMT by increasing the expression of the epithelial markers E-cadherin and cytokeratin 18 and by reducing expression of the mesenchymal marker vimentin, compared to untreated everolimus-resistant HCC cell lines, demonstrating the capability of vitamin D to directly act in the modulation of everolimus resistance by reverting the mesenchymal-like phenotype to an epithelial-like phenotype [25]. Moreover, the detailed examination of liver miRNAs expression revealed the capability of vitamin D to regulate drug resistance by inducing indirect effects. Indeed, everolimus resistant HCC cells exposed to vitamin D showed a significant downregulation of miR-375 that triggered, in turn, a decreased expression of several oncogenes, such as metadherin, YAP-1 and c-MYC proteins, particularly involved in cell proliferation and cancer drug resistance [25]. Moreover, as demonstrated in multidrug resistant leukemic cell lines, vitamin D exposure may reverse drug resistance in a dose-dependent manner by inhibiting multidrug resistance protein 1 (MDR1) and multidrug resistance-associated protein 1 (MRP1) *P-*glycoproteins messenger expression. Furthermore, the same treatment schedule with vitamin D strongly affects intracellular content of glutathione, demonstrating that the vitamin D-mediated drug-resistance reversal mechanism depends on MDR1 and MRP1 *P-*glycoproteins function [26]. Similarly, in a multidrug resistant variant of human small cell lung cancer cell line NCI-H69, calcitriol and its analog calcipotriol, displayed toxicity for MRP1 *P-*glycoprotein expression, thus inhibiting its transport function. As demonstrated by vesicular transport studies through FRET-based high throughput screening, calcitriol and calcipotriol caused a loss of mitoxantrone and doxorubicin transport and inhibited the uptake of tritium-labeled estradiol glucuronide and leukotriene C4 in this cell model, by demonstrating that vitamin D may modulate multidrug resistance process by regulating *P-*glycoproteins transport activity [27].

Taken into consideration the few available molecular data, it can be concluded that vitamin D may act on multidrug cancer resistance at different levels. The studied molecular mechanisms involve EMT transition and cancer stem cells inhibition. Moreover, the role of vitamin D on modulation of miRNA expression and, subsequently, on oncogenes regulation, could be a promising field for future investigation.

## 9. Conclusions

In conclusion, the review of scientific literature concerning the role of vitamin D in the risk of developing colorectal, prostate and breast cancer from 2010 to 2020 has revealed that although there are still conflicting data in this field, a pivotal role of this hormone in cancer progression and mortality exists. Molecular scenario induced by vitamin D in cancer context is traditionally linked to its antiproliferative and apoptotic effects, exerted also in combination with different drugs in order to empower medical treatment. Notable evidence reported by studies on animal and cell culture models also highlighted the importance of its antiinflammatory role in cancer.

Last, despite the few works present in the literature, new compelling evidence about the role of vitamin D in the reversion of multidrug resistance is emerging, showing that through miRNA and EMT regulation vitamin D may overcome the increasingly widespread mechanisms of drug resistance in the oncological field, and suggesting its use in the therapeutic approaches for targeting tumors with the multi drug resistance phenotype.
